# Supplementary material for: Deep inference of seabird dives from GPS-only records: Performance and generalization properties
Source: PLoS Comput Biol. 2022 Mar 11;18(3):e1009890. doi: 10.1371/journal.pcbi.1009890 (PMC8942281; doi:10.1371/journal.pcbi.1009890)
Supplement: Attachment — Submitted filename: review_letter.pdf [file pcbi.1009890.s001.pdf]

Authors' Response to Reviews of

Deep learning and Trajectory Representation for the Prediction of Seabird Diving Behaviour

A. Roy, S. Lanco Bertrand, R. Fablet
PLOS Computational Biology, PCOMPBIOL-D-21-00564

RC: ***Reviewers' Comment***, AR: Authors' Response, L. XX Lines in Revised Manuscript

Dear editor, dear reviewers,

We would like to thank you for your constructive comments. We have been careful to take into account each of the remarks, which has led us to extensively revise our manuscript. One of the main concerns was about the advantages of the Distance Matrix Encoder (DME) network. As suggested, we tested and realized that simply using time-series of speed, bearing, and coverage as the input of U-Network was enough to achieve good performance comparable to the DME-UNet. We are therefore truly grateful to reviewers who helped us to get a deeper and more precise evaluation of our protocol of analysis. As a consequence, we removed all analysis concerning the DME, and we have focused one other important remarks concerning the generalization properties of deep networks. With an additional dataset, we investigated further the ability of deep networks to transfer knowledge acquired from one dataset to another. We also tested the performance of deep networks in response to the amount of data, and we illustrate how to take benefit of pre-trained networks through a fine-tuning procedure. We share our trained models on our github repository so that they could be a relevant basis for future studies dedicated to other trajectory data, possibly beyond seabird datasets. We do think that this review has considerably improved the content and quality of our paper.

Hereafter you will find detailed response to each comment.

Best regards,

Amédée Roy et al.

Reviewer #1

L. 19 & 27

RC: The term "megafauna" is confusing and misused since it is commonly only for large (in size) fauna, such as large herbivores, whales etc. Seabirds are definitely not megafauna. A better term for your study would be top marine predators.

AR: As suggested we have replaced the occurences of "megafauna" with "top predators" L. 37

L. 27

RC: To be good indicators they need to be both sensitive and changing in a predictable manner.

AR: As suggested we removed the sentence describing good indicators.

L. 32-34

RC: I certainly do not agree with the statement of these 2 sentences. At-sea predation do exist and by-catch as well; they are many papers showing how fishing vessels can be tracked by seabirds and also be negatively impacted (by-catch and easy food). In addition, since they are breeding they foraging is done to secure prey for their brood and this is clearly a constraint on their movements; for instance most penguins do long range movements for their chicks and short and local movements at-sea for their own needs. Please correct the parag accordingly.

AR: We agree that it was a shortcut from our side. For this reason we remove these sentences, and focused instead on the importance of dives' distribution in seabird ecology L. 44

L. 87

RC: The authors mentioned that they interpolated missing data. How many were missing? Testing the impact of interpolation on the final analyses is advised.

AR: We refer to missing data as 'gaps', and the amount of gaps is detailed on Table 3. We changed the description of this point for more clarity. We also added a sentence in the text for referring to Table L. 104. Concerning the linear interpolation, it may indeed have an impact on the quality of dives estimation, as for linearly-interpolated gaps, turning angle is zero and step speed is constant. As suggested, we discussed the robustness of the benchmarked approaches with respect to the linearly-interpolated gaps. Especially, our results suggest that neural networks are more robust than HMMs in interpreting these gaps in the data L. 269.

L.93

RC: Although the splitting % is fine and quite standard for deep learning, it does not give much replicates, which is critical for these data hungry approaches. Seventy % of 234 foraging trips, is 163.8, 20% is 46.8 and 10% is 23.4. Since sample size limit is a function of the complexity of your model (and yours is certainly one), it would be appropriate to quantify the performance of your DL algorithm in response to the amount of data (many models with similar complexity require > 1000 of replicates and to me, the foraging trips are the sample size as the location points within them are not independent). Not only this will help show the readers that your approach is robust, but it will serve as a benchmark for others in the future who may have a different number of foraging bouts. Often in ecological studies, researchers do not have the means to equip that number of individuals so this can help having other using your approach in the future with their own (limited) dataset.

AR: This is a very interesting remark. Supervized learning techniques are known to require a lot of data, whereas ecological dataset are often relatively small. However, for seabird diving prediction the deep network we used does not require hundreds of trajectories to converge. We quantify the performance of deep learning algorithm in response to the number of trips in an additional figure Figure 7. We show that for training a deep network from scratch a training dataset of about 30 trajectories is enough (i.e. in our case 20k to 50k GPS positions). For this reason, the splitting that we used provides enough replicates in the training dataset. However, we could argue that the testing datasets might not capture all data variability when using only 10% of trips. Thus we changed the splitting and we used respectively 50%, 30% and 20% of the trips for training, validation and test datasets.

Discussion

RC: **A dedicated parag on the ecological aspects of the datasets and the consequences and potential applications for other types of data is warranted.**

AR: We added a paragaph at the beginning of the discussion where we discuss the different foraging strategies of the seabird that we studied L. 249. Besides the illustration of fine-tuning techniques to take advantage of pre-trained models we discuss the potential application of these deep networks for segmenting other seabird trajectories L. 316.

Editorial issues:

RC: **The numbering of the references is all wrong; they are not cited in order; e.g. you start by citing ref [2] L.20 then ref [20] L.22 etc.**

AR: This has been fixed.

Reviewer #2

RC: **The input of UNet (Fig.3) is time-series of longitude, latitude, and coverage. However, the longitude and latitude are meaningless to detect diving events. To recognize events of moving objects, speed and bearing (angle) are usually used. I consider that when the authors simply use time-series of speed, bearing, and coverage as the input of UNet, the method can achieve good performance comparable to DME-UNet.**

AR: As detailed in the first part of this letter, with further analysis we concluded that you are right. For this reason, we removed all results and discussion concerning this UNet, and we focus the analysis to models that take as input the time-series of speed, bearing, and coverage. Thanks a lot for this crucial comment.

RC: **I'm also afraid that the contribution of DME is limited because, as shown in the right graph of Fig.3, the performances of DME-UNet and UNet are similar. Can you make this graph using the cormorant data?**

AR: Idem

1.

RC: **It is good to investigate the contribution of DME deeply. As mentioned above, please use speed and bearing speed (radian per time unit) as additional inputs of UNet. Please also make a graph like Fig 3 using the other test data sets.**

AR: Idem

2.

RC: **The authors try to detect diving events using only GPS data (without using water depth sensor and accelerometer). However, the motivation is not described in the introduction section.**

AR: When external data is available, there is no need to use our approach. However, there is a substantial amount of tracking datasets that consist in GPS tracks only. The development of tools dedicated to animal trajectories

segmentation (i.e. for dive identification) is therefore needed to extract more out of these datasets. We clarified this motivation in the introduction part L. 52.

3. Line 84

RC: How to extract foraging trips from GPS records? Please explain.

AR: We added in the material and method part a short description of the procedure used to extract foraging trips. It simply consisted in selecting locations further than a given distance to the colony and longer than a given time L. 100

4. Line 85

RC: How did you synchronize time stamps of GPS and TDR?

AR: Except from interpolating GPS data at the TDR sampling resolution, we have not undertaken any additional pre-processing concerning GPS and TDR synchronization. We clarified this point L. 104

5. Table 1

RC: Gaps in Table 1 is not explained.

AR: Gaps consist in the amount of missing data. We detailed it in the table caption Table 1

6.

RC: The authors use AUC to evaluate the methods. However, the goal of the authors is detect diving events. So, it is better to show the classification performance of the proposed method (e.g., F1-score of diving).

AR: We agree with the reviewer that the F1-score may also provide a relevant performance metrics. We added it to result tables Tables 2 & 3. It however focuses on a single detection threshold. By contrast, ROC AUC provides a more comprehensive evaluation of the segmentation performance through the joint analysis of the dive detection performance vs. the false detection rate. Especially, depending on the targeted scientific questions or applications, one may expect to optimize the detection of diving or non-diving behaviours. In such cases, a single F1-score may not be highly informative to discriminate approaches.
